# The Hemolymph Proteome of Fed and Starved *Drosophila* Larvae

**DOI:** 10.1371/journal.pone.0067208

**Published:** 2013-06-20

**Authors:** Björn Handke, Ingrid Poernbacher, Sandra Goetze, Christian H. Ahrens, Ulrich Omasits, Florian Marty, Nikiana Simigdala, Imke Meyer, Bernd Wollscheid, Erich Brunner, Ernst Hafen, Christian F. Lehner

**Affiliations:** 1 Institute of Molecular Life Sciences (IMLS), University of Zurich, Zurich, Switzerland; 2 Department of Biology, Institute of Molecular Systems Biology (IMSB), ETH Zurich, Zurich, Switzerland; Shantou University Medical College, China

## Abstract

The co-operation of specialized organ systems in complex multicellular organisms depends on effective chemical communication. Thus, body fluids (like blood, lymph or intraspinal fluid) contain myriads of signaling mediators apart from metabolites. Moreover, these fluids are also of crucial importance for immune and wound responses. Compositional analyses of human body fluids are therefore of paramount diagnostic importance. Further improving their comprehensiveness should increase our understanding of inter-organ communication. In arthropods, which have trachea for gas exchange and an open circulatory system, the single dominating interstitial fluid is the hemolymph. Accordingly, a detailed analysis of hemolymph composition should provide an especially comprehensive picture of chemical communication and defense in animals. Therefore we used an extensive protein fractionation workflow in combination with a discovery-driven proteomic approach to map out the detectable protein composition of hemolymph isolated from *Drosophila* larvae. Combined mass spectrometric analysis revealed more than 700 proteins extending far beyond the previously known *Drosophila* hemolymph proteome. Moreover, by comparing hemolymph isolated from either fed or starved larvae, we provide initial provisional insights concerning compositional changes in response to nutritional state. Storage proteins in particular were observed to be strongly reduced by starvation. Our hemolymph proteome catalog provides a rich basis for data mining, as exemplified by our identification of potential novel cytokines, as well as for future quantitative analyses by targeted proteomics.

## Introduction

Extracellular body ﬂuids, for example blood plasma or interstitial fluid, serve as transport systems for metabolites, nutrients, hormones or oxygen in virtually all animals. Insects have only one extracellular ﬂuid called hemolymph that is usually kept in circulation by an open heart within the body cavity. The hemolymph is in direct contact with all internal organs. It delivers necessary substances such as nutrients to the cells and it transports metabolic waste products away from those same cells. It contains hemocytes, most of which are phagocytic cells [1]. Moreover, it contains proteins that provide hemostatic responses to wounding [2]. Many additional hemolymph proteins help to protect the insect against invading microorganisms [3]. Hormones that regulate developmental timing, metamorphosis, metabolism, growth, reproduction and associated behavior are secreted and circulated in the hemolymph [4], [5], [6], [7], [8].

Despite its importance for development and physiology, there is only limited information about insect hemolymph composition. Initial biochemical analyses have focused on low-molecular-weight compounds such as inorganic salts, amino acids, organic acids, lipids and sugars [9], but not much is known about the protein composition of hemolymph. Mass spectrometry-based proteomics (MS) has emerged as a powerful tool for monitoring protein composition in body ﬂuids in different states. Studies in several insect species have identified hemolymph proteins after separation by one- and two-dimensional gel electrophoresis in conjunction with MS [10], [11], [12], [13], [14], [15], [16], [17], [18], [19], [20], [21], [22], [23]. More comprehensive proteomic analysis including in-solution digestion of hemolymph proteins followed by liquid chromatography-mass spectrometry (LC-MS) has been carried out for the honey bee [24] but is prominently missing for the fruit fly *Drosophila melanogaster*, the insect model system most widely used in cell and developmental biology that has provided invaluable insights of very general significance for eukaryotes including humans.

Elegant co-culture experiments with isolated organs from *Drosophila* larvae have strongly suggested that starvation affects not just the metabolite [25], [26], [27] but also the signaling factor composition of hemolymph [28], and recent genetic analyses have clearly identified secreted signaling proteins that are thought to have a variable hemolymph concentration regulated by the nutritional status [29], [30], [31]. Therefore, a comparison of hemolymph samples from fed and starved larvae might in principle also point to novel candidate signaling factors although their often very low effective concentrations represents a great challenge.

Here we present a deep shotgun proteomic analysis of hemolymph samples from third-instar *Drosophila* larvae. The overall aim of this study was to establish a comprehensive proteome map [32] of the *Drosophila* larval hemolymph. In addition, we describe an initial step towards an understanding of the impact of nutrient conditions on hemolymph protein composition. Our results extend the number of known hemolymph proteins by almost an order of magnitude and demonstrate dramatic starvation effects on storage proteins.

## Materials and Methods

### *Drosophila* Culture and Hemolymph Isolation

Flies of the *Oregon R* (*OreR*) wild-type strain were cultivated at 25°C and 45% relative humidity on standard food (100 g/l yeast, 75 g/l sucrose, 55 g/l cornmeal, 10 g/l wheat flour, 8 g/l agar, 0.45 ml/l nipagine, 0.9 ml/l propyl paraben). To generate larvae for hemolymph collection, an initial egg collection for the elimination of overaged eggs was performed during 1 hour in fresh fly bottles with standard food. Thereafter, flies were transferred to another set of fresh fly bottles with standard food and eggs were collected for 2 hours. Flies were discarded and the bottles with the eggs were incubated at 25°C. After incubation for 64 hours, larvae were washed out from the food and transferred to either bottles with fresh standard food (fed) or to bottles with filter paper soaked with 20% sucrose (starved). Hemolymph isolation was started after incubation for another 24 hours at 25°C. Immediately before hemolymph collection, larvae were surface sterilized in 70% ethanol. Excess fluid was blotted off on filter paper. Batches of 10–15 larvae were dipped in halocarbon oil of high viscosity (Halocarbon-oil 1000N, Solvadis Chemag, #102780) and opened by gently pulling the epidermis apart with forceps to start hemolymph bleeding. The hemolymph accumulated in a drop around the larvae was collected with a fine glass pipette, transferred into an Eppendorf tube and immediately frozen in liquid nitrogen. We emphasize that our isolation procedure did not include removal of hemocytes in order to minimize the time available for potential proteolytic and chemical modifications of hemolymph proteins during isolation that harm subsequent proteomic analyses. The complete sampling procedure until freezing took less than two minutes and resulted in 3–4 µl of hemolymph per aliquot. Between 30–40 aliquots, collected from three independent batches of larvae, were pooled for the MS analyses that resulted in the data described in Table S1. Pools of around 20 additional aliquots collected from independent batches of larvae were used in an initial pilot MS analysis. Developmental stages of larvae were assigned based on mouth hook morphology [33].

### Sample Preparation for Mass Spectrometry

Pooled hemolymph aliquots were briefly centrifuged for removal of insoluble material and lysed in 50 mM (NH_4_)HCO_3_ containing 0.2% RapiGest (Waters). The protein concentration was determined in a Qubit fluorometer (Invitrogen). 1 mg of the total protein lysate was reduced with 5 mM Tris(2-carboxyethyl)phosphine hydrochloride (TCEP) and treated with 10 mM iodoacetamide to modify cysteine residues. Tryptic digestion was carried out overnight using 20 µg trypsin (Promega) per sample and a concentration of RapiGest of 0.1%. The samples were purified by reverse phase C-18 chromatography (Sep-PaK, Waters). For sample fractionation, isoelectric focusing of peptides was performed (OFFGEL fractionator 3100, Agilent). A 24 well strip with a linear pH gradient ranging from 3–10 was used (GE Healthcare). The offgel (OG) fractionation was performed as described [34]. In short, the OG fractionation was started after dispensing 150 µl of the peptide solution in each well. The potential was fixed for the first hour at 500 V, then set to a maximum of 8000 V and after finishing the separation kept at 500 V (total of 50 kVh; total run time ∼18 h). The current limit was set at 100 µA and the temperature was maintained at 20°C. After OG fractionation, the 24 peptide fractions were cleaned by reverse phase C-18 chromatography (MicrospinColumns, SEM SS18V, The Nest Group, Inc).

### Mass Spectrometry Analysis

For mass spectrometry analysis samples were resuspended in 50 µl of buffer A (5% acetonitrile, 0.2% formic acid). From each sample, 1 µl of material was loaded on a LTQ-Orbitrap XL ETD (Thermo Fisher Scientific). The instrument was coupled to an Eksigent nano-LC system. Samples were automatically injected into a 10-µl sample loop and loaded onto an analytical column that was packed in-house with Magic C18 AQ beads (3 µm, 100 Å, Microm) 9 cm in length × 75 µm (internal diameter). Peptide mixtures were delivered to the analytical column at a flow rate of 500 nl/minute (3% acetonitrile, 0.2% formic acid) for 16 minutes and then eluted using a gradient of acetonitrile (3%–35%; 0.53%/minute) with 0.2% formic acid at a flow rate of 250 nl/minute. The samples were measured in a survey scan from 300 to 2,000 a.m.u., followed by 6 data-dependent MS/MS scans with dynamic exclusion (isolation width 2 a.m.u., repeat count 1, exclusion list size 500, dynamic exclusion duration 60 s). In a second survey scan, the same settings were applied with the addition of a static exclusion list of all peptides monitored in the first survey scan. The static exclusion list contained all MS1 spectra with an assigned MS/MS from the first survey run. Subsequently, the first two survey scans were used to generate a third survey scan with inclusion lists for the MS1 features which had not been analyzed by either the first or the second MS run. The inclusion lists for all OG fractions were generated with the Progenesis software tool (Non Linear Dynamics, New Castle upon Tyne, UK Version 4.0). Manually, seeding vectors (4–7) were set over the whole retention time followed by automatic alignment of the feature maps with a sensitivity threshold of 3. Filters for features with MS/MS were applied to remove those. The remaining MS1 features were exported with a retention time window of 2.5 min to an Xcalibur compatible inclusion list. The generated inclusion list was used to perform a third survey scan on the respective OG fraction. In total, 144 measurements were performed.

### Database Search and Protein Identification

Raw data were converted into the open format mzXML. Using the Sequest algorithm [35], fragment mass spectra were searched against a protein sequence database containing 21,317 *D. melanogaster* proteins (FlyBase version 2008_10) and 256 common contaminants (keratins, trypsin, etc.). Spectra were searched for a match to fully-tryptic and semi-tryptic peptides with up to two missed cleavage sites with a mass tolerance of 0.04 Da. Carbamidomethylation (+57.021464 Da) was set as fixed modification for all Cysteines and oxidation (+15.994915 Da) was considered as optional modification for Methionines. Search results were post-processed using Peptide Prophet (TPP version 4.5.0) [36] to model correct versus incorrect peptide spectrum matches (PSMs). Based on the target-decoy search strategy [37] a stringent score cutoff was determined that resulted in an estimated false discovery rate (FDR) of less than 0.2% at the PSM level. PSMs above this cutoff were classified with the PeptideClassifier software [38].

A minimal list of unambiguous protein identifications (based on class 1a, 1b, or 3a peptides) and protein group identifications that imply one gene model (based on class 2a, 2b peptides) was generated (Table 1). For class 3b peptides, which imply distinct proteins encoded by different gene models, the minimal possible number of protein groups not identified by peptides of higher information content was determined. For a protein identification, we required at least two independent PSMs. This resulted in a final estimated protein-level FDR of 1.3%. Raw data from the proteomic experiments will be made available at PRIDE (http://www.ebi.ac.uk/pride/).

**Table 1:** Summary of identified spectra, peptides, proteins and estimated FDR levels.

Evidence classa)	No. of spectra	No. of distinct peptides	No. of distinct proteinsb)
Class 1a	51,359	4,129	429
Class 1b	5,005	784	117
Class 2a	1,282	170	25
Class 2b	8,053	1,377	120
Class 3a	159	23	6
Class 3b	3,631	251	28c)
target DB	69,489	6,734	725
decoy DB	128	55	10
estimated FDRd)	<0.2%	<0.8%	<1.4%

a) According to our peptide classification scheme [38], [46], class 1a peptides unambiguously identify a single unique protein sequence encoded by a unique transcript. Class 1b peptides also unambiguously identify a unique protein sequence encoded by several transcripts of the same gene model with identical coding region and differences in the 5′ and/or 3′ untranslated regions. Class 2a peptides identify a subset and class 2b peptides all protein sequences encoded by a gene model. Class 3a peptides unambiguously identify one protein sequence, but this sequence could be encoded by several gene models from distinct loci (e.g. histones). Finally, class 3b peptides can be derived from different protein sequences encoded by several gene models from distinct loci and have the least information content.

b) For protein groups identified by class 2a or 2b peptides (a gene model identification) all possible protein accessions are listed in Table S1.

c) The minimal number of additional protein identifications by 3b peptides is shown.

d) Based on the total hits in target and decoy databases (DB), the FDR was estimated at the spectra, peptide and protein level.

For prediction of globular proteins we used Globplot 2.3 (http://globplot.embl.de/) [39], for prediction of signal peptides SignalP 4.1 (http://www.cbs.dtu.dk/services/SignalP/) [40].

### Differential Protein Expression Analysis

Using the decoy-search hits, peptide-spectrum matches (PSMs) were stringently filtered to a FDR of less than 0.2%. Corresponding peptides were classified with the PeptideClassifier software [38]. A minimal list of unambiguous protein identifications (based on class 1a, 1b, or 3a peptides) and protein group identifications that imply one gene model (based on class 2a, 2b peptides) was generated (Table 1). For class 3b peptides, which imply distinct proteins encoded by different gene models, the minimal possible number of protein groups not identified by peptides of higher information content was determined.

Differential protein expression analysis was carried out with the R package DESeq (version 1.6.1) [41]) using the spectral count data as input. Based on normalized count data, DESeq modeled gene/protein expression with a negative binomial distribution and generated a list of genes/proteins ranked according to statistical significance. Default parameters were chosen as described in the DESeq package vignette (a “local” fit was used to estimate dispersion).

## Results and Discussion

**Figure 1:**
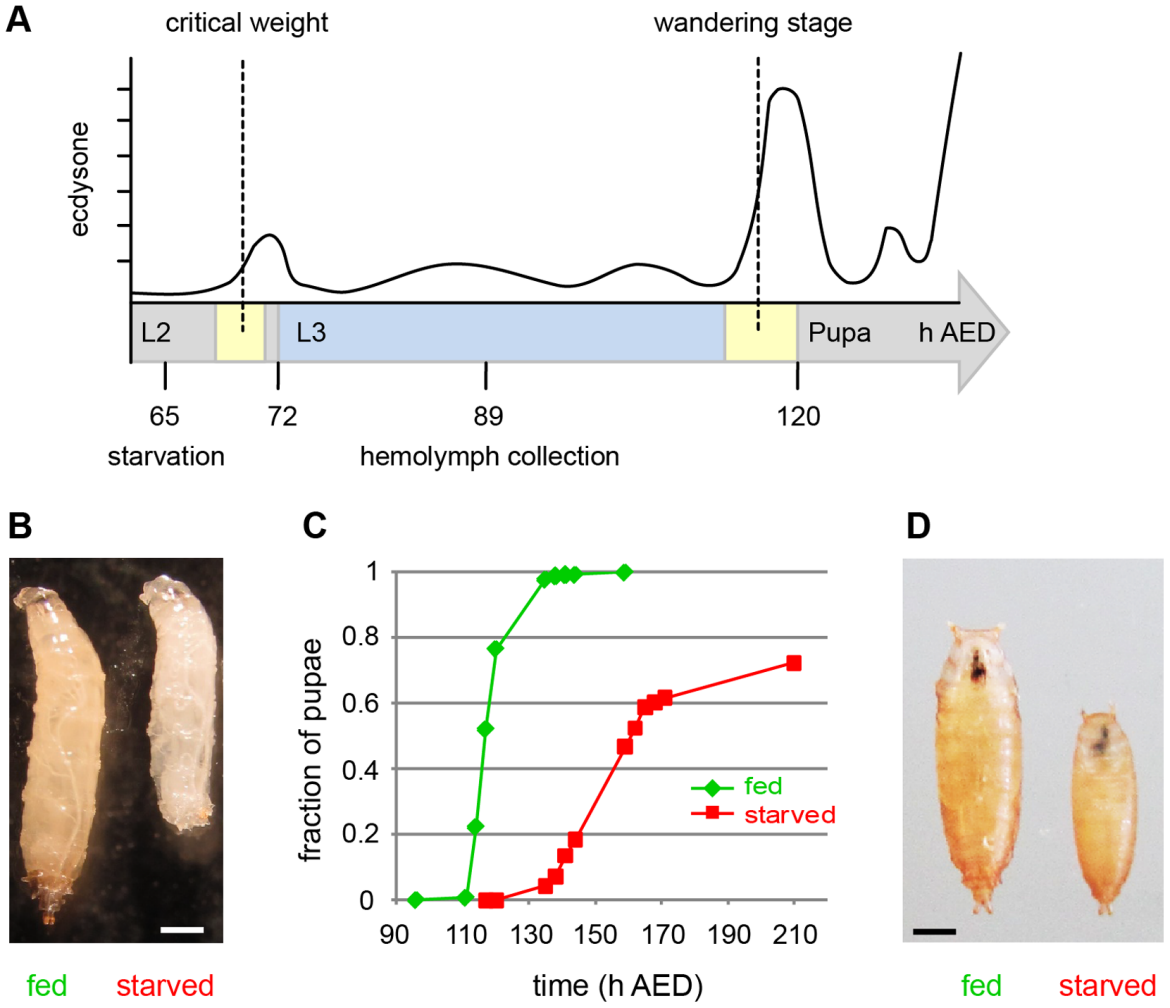
Starvation protocol and developmental effects. (A) At 65 hours after egg deposition (AED), half of the larvae were transferred to starvation medium (20% sucrose). Twenty-four hours later, hemolymph from fed and starved larvae was collected for deep shotgun proteomics. Developmental timing of ecdysone titer, larval stages L2 and L3, acquisition of critical weight, wandering behavior and pupation under optimal conditions is indicated as well. Numbers indicate time in hours AED. (B) Size of fed and starved larvae at time of hemolymph collection. (C) At 65 hours AED, larvae were either shifted to starvation medium or further maintained on rich medium followed by analysis of the fraction of pupae over time (n = 278 fed and 141 starved) (D) Size of pupae formed by either fed or starved larvae. Bars = 0.5 mm.

Hemolymph was collected from larvae of the *Oregon R* wild-type strain. A first batch was isolated from larvae obtained after egg collection for 2 hours and ageing for an additional 89 hours at 25°C in the presence of unlimited standard *Drosophila* food (Fig. 1A). At the time of hemolymph isolation these larvae were therefore expected to be in mid L3 stage. Scoring of larval mouth hook morphology, which allows accurate larval stage assignment, clearly confirmed that the larvae had all reached the L3 stage (n = 50). Mid L3 was chosen for hemolymph collection as this stage is accompanied by the most extensive growth of all *Drosophila* development [33]. For comparison, we also analyzed hemolymph from larvae of identical age after exposure to starvation conditions (Fig. 1A). During the last 24 hours, this second batch of larvae was aged in the presence of 20% sucrose, i.e. without a source of amino acids and other non-carbohydrate metabolites. At the onset of starvation, the majority of larvae were still in the L2 stage according to mouth hook morphology (62.9% in L2, 5.6% during L2/L3 molt, 31.5% in L3; n = 54). Later, at the time of hemolymph isolation, all the starved larvae had reached the L3 stage (n = 50) but they were clearly smaller than the fed larvae (Fig. 1B). Moreover, larvae that were kept further under starvation condition instead of being sacrificed for hemolymph collection did not pupariate like the fed larvae (Fig. 1C). Pupariation was either blocked (in ∼30%) or delayed (in ∼70%). The pupae formed by the starved larvae were smaller than those of fed larvae (Fig. 1D). These results confirm that starvation was initiated at a time when the majority of the larvae had not yet reached the so-called critical weight. Starvation before attainment of the critical weight is known to delay metamorphosis onset, while later starvation no longer causes delays [42].

**Figure 2:**
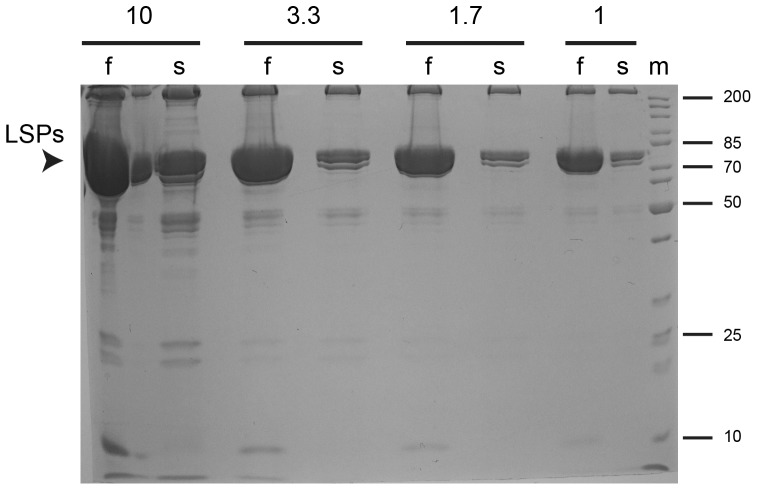
Abundance of larval serum proteins. Hemolymph was isolated from fed (f) and starved (s) larvae (see Fig. 1). Proteins in samples of 10, 3.3, 1.7 or 1 µl hemolymph were resolved by SDS-PAGE and stained with Coomassie Blue. The position of the major larval serum proteins (LSPs) is indicated by an arrowhead. Position and size (kDa) of molecular weight markers (m) are indicated on the right side.

For the isolation of hemolymph, larvae were gently opened with forceps to release undiluted hemolymph that was quickly isolated without removal of hemocytes. Compared to fed larvae, protein concentration in hemolymph isolated from starved larvae was found to be about twofold lower in two independent experiments. Analysis by SDS-PAGE revealed that hemolymph of starved larvae contained far lower levels of the predominant hemolymph proteins with apparent molecular weights around 80 kDa (Fig. 2). These larval serum proteins (Lsp1α, Lsp1β, Lsp1γ, and Lsp2) are strongly up-regulated during the L3 stage. Their amount in hemolymph of third instar wandering stage larvae grown in rich medium corresponds to up to 70% of the total hemolymph protein [43], [44], [45].

**Figure 3:**
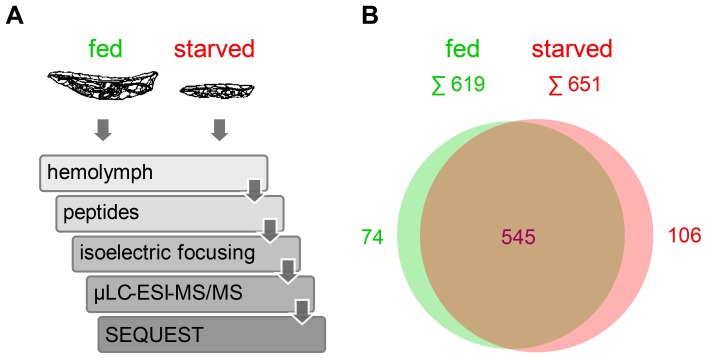
Characterization of the larval hemolymph proteome. (A) Workflow of the analyses. Hemolymph samples from fed and starved larvae were digested in solution. Tryptic peptides were separated by isoelectric focusing for complexity reduction. Peptides were analyzed using microcapillary liquid chromatography–electrospray ionization–tandem MS (µLC-ESI-MS/MS). SEQUEST spectral search was performed for peptide spectrum matching. (B) Venn diagram illustrating the number of gene models detected in hemolymph from fed and starved larvae, respectively.

Hemolymph samples were analyzed on an Orbitrap XL mass spectrometer (Fig. 3A). Peptides were classified using a deterministic classification scheme [38], [46] (Table S1). Within the two samples, we identified in total 6734 unique peptides corresponding to protein products from 725 different gene models with a FDR of 1% (Table 1, Fig. 3B). 75% (545 gene models) were detected in hemolymph from both fed and starved larvae. 10% (74 gene models) were only detected in hemolymph from fed larvae, while 25% (106 gene models) were only observed in hemolymph from starved larvae in which also a higher total number of different gene models were detected (651 versus 619). Previous analyses of the *Drosophila* hemolymph proteome [11], [12], [14], [16], [17], [18], [47] have been considerably less comprehensive. Overall these earlier studies have detected only 13% of the gene models identified in our analysis. 90% of the previously identified hemolymph proteins were also detected in our study. The large majority of these previously described proteins are very abundant hemolymph components as inferred from spectral counting [48] (Table S2). Similarly, previous analyses of the hemolymph proteome in other insects (including the bee *Apis mellifera*, the silkworm *Bombyx mori*, and the tobacco hornworm *Manduca sexta*) have been of comparatively limited scope, revealing primarily abundant constituents [10], [15], [19], [24], [49], [50], [51], [52], [53], [54].

The substantial increase in the number of identified hemolymph proteins resulting from our analysis in *Drosophila* larvae brings the complexity of this proteome far closer to the range described for the extensively analyzed human plasma proteome. The human plasma proteome project has detected a highly non-redundant set of 1929 protein sequences at 1% FDR [55]. In both, human plasma and *Drosophila* larval hemolymph, protein abundances vary over a very wide range. The concentration (∼40 mg/ml) of the most abundant protein in human plasma, serum albumin, is comparable to that of the most abundant component of *Drosophila* larval hemolymph, the major apolipoprotein Rfabg [56], [57]. By intense shotgun proteomics, proteins with a concentration more than 6.5 orders of magnitudes lower have been identified in human plasma. Nevertheless, shotgun proteomics has clear limitations especially in case of low abundance proteins. Some human plasma proteins are known to have concentrations that are more than 10 orders of magnitude lower than the most abundant components, and in general, the known low abundance proteins have escaped detection by shotgun proteomics [55], [58]. Moreover, in this approach low abundance is just one of several limiting factors with protein size and absence of suitable tryptic cleavage sites being among the additional crucial detection determinants. Thus our protein catalog of *Drosophila* larval hemolymph cannot be expected to be complete and an absence of some known hemolymph constituents is clearly evident. For example, we have not detected insulin-like peptides encoded by the *dilp* genes. Dilps 2, 3 and 5 are released into the hemolymph from specialized neurosecretory cells within the larval brain in response to nutrient uptake and presumably act at nanomolar concentrations [30], [31]. Similarly, we did not detect Upd2/Leptin that is secreted from the fat body in response to nutrient uptake and triggers Dilp 2/5 release from the brain neurosecretory cells [29]. Detection and quantification of very low abundance components will require different and targeted approaches [32], [59], [60], [61]. Moreover, depletion of quantitatively dominating components is an additional strategy allowing deeper sampling. As *Drosophila* Lsp null mutants are viable and fertile [45], analyses of their hemolymph might further increase overall proteome coverage in future studies.

In contrast to Dilps and Upd2, we have readily detected other proteins that have been proposed to function as growth factors. For example, we have clearly observed the products from all of the six *Drosophila* members of the family of Imaginal Disc Growth Factors genes (*Idgf1-5*, *CG5210*). In fact, our data suggests that these chitinase-related proteins are abundant hemolymph components (among top 10%) in fed and in starved larvae. IDGFs were originally identified in conditioned medium because of their growth-promoting activity on *Drosophila* cl8 cells [62], [63]. Moreover, we also detected Adenosine deaminase-related growth factor A (Adgf-A) in hemolymph. Adgf-A is the main regulator of extra-cellular adenosine during larval stages and has been shown to play important roles in the control of hemocyte proliferation [64], [65], [66].

Our hemolymph proteome contains several proteins that have not been detected in previous shotgun analyses but were recently shown to be hemolymph proteins after an initial identification by genetic approaches while our work was ongoing. The minor apolipoproteins apoLTP/CG15828 and Cv-d/CG31150 [57], [67] belong to this group for example.

To illustrate the potential of our hemolymph protein catalog for data mining and future functional analyses, we generated a list of potential novel cytokines (Table S2). For this list, we filtered out all CG numbers that were predicted to encode a globular secreted protein smaller than 400 amino acid residues. Moreover, we retained only those that have not yet been reported to be hemolymph components according to our knowledge. The resulting list comprised 30 entries that might deserve further analysis. Two among this list (CG15201 and CG31997) are SVC family proteins that have a motif initially proposed to be related to insulin-like growth factor (IGF) but more recently classified as more similar to the C-domain of von Willebrand factor (VWC) [68].

As our hemolymph isolation procedure did not include hemocyte removal, detection of some cytosolic and nuclear proteins was expected. Moreover, as larval wounding was involved in our hemolymph isolation procedure, tissue damage and consequential rupture of crystal cells might have augmented a release of non-secreted cellular proteins like histones and ribosomal proteins into the hemolymph [69], [70], [71]. To what extent such release occurs even during unperturbed development of *Drosophila* larvae is not known. Non-secreted cellular proteins detected in our work might therefore have originated from the included intact hemocytes, as well as from lysis of hemocytes and other cells before or during hemolymph isolation. Reliable clarification of the origin of non-secreted cellular proteins will require additional experiments and will depend on methods with detection sensitivity higher than shotgun proteomics in particular in case of those revealed by only one or a few peptides. A provisional estimate based on our histone peptide counts and PaxDb data concerning humans [72] suggested that tissue leakage into our hemolymph samples has occurred to a comparable extent as apparent in case of human plasma. Moreover, assuming that our 24 hour starvation period did not have significant effects on cellular levels of ribosomal proteins, the numerical comparison of all unequivocal peptides derived from ribosomal proteins (297 in fed, 264 in starved) suggests that the release of non-secreted cellular proteins into our two samples has occurred to a comparable extent.

**Figure 4:**
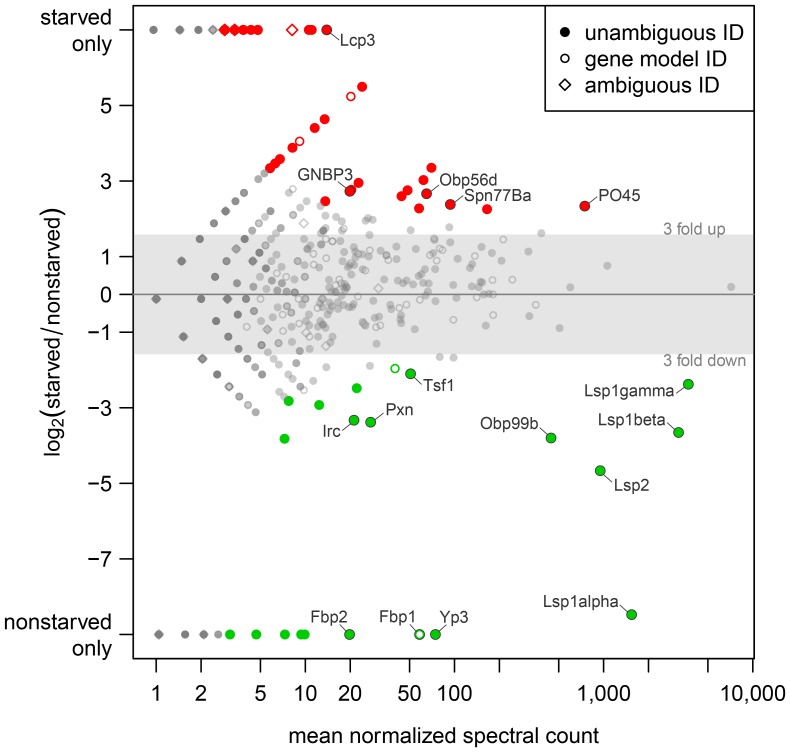
Effects of starvation on hemolymph proteome. The magnitude versus amplitude (MA) plot shows the log2 fold change of the expression of the identified *D. melanogaster* proteins in the starved versus fed condition against the mean normalized spectral count. The top 10% differentially expressed proteins are highlighted, including 50 up-regulated proteins (red dots) and 22 down-regulated proteins (green dots). Protein identifiers are shown for selected proteins discussed in the text. Unambiguous protein identifications by class 1a, 1b, and 3a peptides are shown as full circles. Protein groups identified by class 2a or 2b peptides (which unambiguously imply a gene model) are shown as open circles, ambiguous identifications by 3b peptides are shown as open diamonds (the respective identifiers are listed in Table S2).

To identify proteins with different abundance in hemolymph from fed and starved larvae, respectively, we compared spectral counts using DESeq [41] (Fig. 4, Table S3). Spectral counting is only an approximate measure of abundance. Moreover, an interpretation of our spectral counts needs to take into account that the total protein concentration in hemolymph from fed and starved larvae is not identical. As indicated above, protein content of hemolymph from starved larvae is twofold lower compared to fed larvae primarily because of the absence of larval serum proteins in starved larvae. As we have analyzed the same amount of total protein for the fed and the starved sample, normalization is not trivial. We emphasize that the differences in protein abundance suggested by our data may not necessarily reflect the reality, in particular in case of proteins with low spectral counts, where sampling bias and contingencies as well as normalization problems might have caused distortions. As a consequence, we restrict our following comments to cases with putative concentration differences that were far more extensive than twofold and also apparent in an independent biological replicate, our initial smaller pilot experiment. For these proteins statistical support for differential abundance was very strong. The top 10% of the differentially regulated proteins resulting from 40 genes are compiled in Table 2 (for complete data set see Table S3).

**Table 2:** Starvation-associated protein abundance changes in larval hemolymph.

gene symbol	after starvation	p-value	total counts	log2 starved/fed	transcript dev. expr.a)	comment
Yp3	Down	1.04E-04	143	-Inf	−3.59694	yolk protein, female specific
Fbp1	Down	2.37E-04	112	-Inf	−14.7243	fat body protein 1
Fbp2	Down	0.004945	38	-Inf	−9.69436	fat body protein 2
CG7320	Down	0.024777	19	-Inf	−6.07039	hexamerin related
CG3264	Down	0.027552	18	-Inf	0.058894	putative alkaline phosphatase
CG31075	Down	0.044311	14	-Inf	−1.07039	putative mito. aldehyde dehydrogenase
Npc2h	Down	0.096736	9	-Inf	−0.926	Niemann-Pick Type C-2h
Lsp1α	Down	4.55E-05	2958	−8.48	−5.40939	Hexamerin
Lsp2	Down	0.006818	1827	−4.67	−7.6886	Hexamerin
CG31769	Down	0.123496	14	−3.82	0.321928	
Obp99b	Down	0.02253	857	−3.80	−8.02791	odorant binding protein
Lsp1β	Down	0.032916	6144	−3.65	−3.88753	Hexamerin
Pxn	Down	0.053156	53	−3.38	−0.1375	Peroxidasin, extracellular matrix
Irc	Down	0.067196	41	−3.33	−0.76553	Immune-regulated catalase
CG13962	Down	0.147981	43	−2.48	−1.20163	
Lsp1γ	Down	0.146536	7195	−2.38	−2.90689	Hexamerin
Tsf1	Down	0.16602	99	−2.10	−1.43296	Transferrin 1
Lcp3	Up	0.013845	29	Inf	−2.26303	Larval cuticle protein 3
CG6180	Up	0.102422	10	Inf	0.321928	putative phosp.ethanolamine bdg. prot.
sPLA2	Up	0.102422	10	Inf	−2	secretory Phospholipase A2
CG13227	Up	0.102422	10	Inf	1.888969	
CG30457	Up	0.102422	10	Inf	3.836501	
Gs2	Up	0.102422	10	Inf	−0.48543	Glutamine synthetase 2
CG6206	Up	0.141877	8	Inf	−0.54597	Lysosomal α-mannosidase
CG6673	Up	0.141877	8	Inf	0.915936	Glutathione S transferase O2
Spn55B	Up	0.141877	8	Inf	−0.28911	Serpin
CG15043	Up	0.141877	8	Inf	0.168123	
Vago	Up	0.169725	7	Inf	−1.66448	single VWC domain protein
Sema-1b	Up	0.169725	7	Inf	0.304006	Semaphorin-1b
CG17278	Up	0.169725	7	Inf	0.514573	
Sap-r	Up	0.169725	7	Inf	−1.65992	Saposin-related
Sp7	Up	0.016934	42	5.24	−0.1375	Serine protease 7, melanization
CG6045	Up	0.043504	28	4.64	−0.18903	
CG10031	Up	0.05937	24	4.40	0.321928	
CG15117	Up	0.09185	19	4.05	1.434403	putative glucuronidase
Cpr51A	Up	0.175735	13	3.47	3.321928	Cuticular protein 51A
GNBP3	Up	0.12615	41	2.73	−1.63691	Gram-negative bacteria bdg. prot. 3
Obp56d	Up	0.076917	134	2.66	−0.22651	Odorant-binding protein 56d
Spn77Ba	Up	0.100789	193	2.38	−0.31034	Serpin 77Ba
PO45	Up	0.1351	1537	2.34	−2.09085	prophenoloxidase 45

a) Change in transcript levels during development in rich medium was estimated based on expression profiling data from [77]. For transcript levels around the time when starvation was started (early) the values observed at L2 and L3/12hours were averaged. For transcript levels around the time of hemolymph collection (late) the values at L3/puff stage 1–2 were used. The given values correspond to log2(early/late).

The lowest p value (p<4.55E-05) resulted in case of Lsp1α where almost 3000 spectra were detected in the hemolymph from fed and only 9 spectra in the sample from starved larvae (Table S3). Strong differences were also observed in case of the closely related Lsp1β, Lsp1γ, and Lsp2. This count data concurs with our independent evidence from the analysis of hemolymph samples by SDS-PAGE and Coomassie Blue staining (Fig. 2). It also agrees entirely with predictions based on the demonstrated developmental delay induced by our starvation protocol (Fig. 1A) and the known developmental regulation of these major Lsps during development in rich medium [44], [45]. Because at the time of hemolymph collection, the starved larvae in contrast to the fed cohort had not yet reached the stage where Lsp expression is maximal, the level of major Lsps is expected to be reduced in the starved larvae. We conclude that in case of the Lsps, spectral counting with our data provided reliable information on abundance.

The *Drosophila* Lsps were originally identified because of their high abundance in hemolymph of third instar wandering stage larvae [73], [74], [75]. They have been shown to serve as storage proteins that are metabolized during the nonfeeding larval wandering and pupal stages (for reviews see [75], [76], [77]. Homologs are found throughout insects and are generally designated as hexamerins since they form homo- and heteromeric hexamers. In preparation for the nonfeeding stages, expression of the *Drosophila* Lsps is strongly induced in the fat body of mid third instar larvae by the raising ecdysone titer. Beyond the traditional focus of attention on this impressive peak of Lsp expression before the nonfeeding late larval and pupal stages recent evidence has suggested that Lsps are also expressed during other stages although at considerably lower levels. Several microarray experiments have clearly demonstrated the presence of in particular *Lsp1β* and *Lsp2* transcripts in adult flies. Moreover, these two genes were among those most strongly and consistently downregulated after 24 hour starvation of adult flies [78], [79]. We propose therefore that these Lsp genes are used for nutrient storage in anticipation of upcoming starvation, not just before the nonfeeding developmental stages where they are regulated by ecdysone, but also during adult life where they appear to be controlled by nutrient availability during cycles of feast or famine. The strongly decreased Lsp abundance in hemolymph of starved larvae might therefore not just reflect an indirect effect of starvation on development but also a more direct non-developmental regulation by nutrients.

Five gene models (*Yp3*, *Fbp1*, *Fbp2*, *CG7320*, *Obp99b*) were identified with characteristic similarities to the major Lsp genes (*Lsp1α, β, γ* and *Lsp2*) (Table 2). The products of these gene models were also absent or dramatically lower in hemolymph of starved larvae. Moreover, according to expression profiling during development in rich medium [80], their transcript levels are strongly upregulated in late third instar larvae coincident with upregulation of Lsp gene expression. Therefore, the strongly reduced hemolymph concentration of the corresponding proteins presumably reflects at least in part the inhibitory effect of starvation on development. However, we point out that transcripts of *Obp99b* were also found to be downregulated strongly and consistently in response to starvation in adults [78], [79], as in case of *Lsp1β* and *Lsp2*. We suggest that the product of *Obp99b*, which is characterized by a developmental transcript profile quite distinct from other related odorant binding proteins [80], might function as a storage protein. In support of this proposal, most of the other gene models (*CG7320*, *Fbp1*, *Fbp2*, and *Yp3*), which with regard to developmental expression profile in rich medium and dependence of protein abundance in hemolymph on larval feeding behave like *Obp99b* and Lsp genes, have close functional connections to storage proteins. *CG7320* encodes an uncharacterized minor hexamerin-related protein. Fat body protein 1 (Fbp1) serves as a receptor for hexamerin re-import into the fat body for production of protein storage granules [81]. Yp3 is a yolk protein known to be used for storage in preparation before the nonfeeding stage of embryogenesis [82].

The other half of the gene models coding for protein products that were absent or decreased in hemolymph of starved larvae (Table 2) did not belong to the group with an *Lsp*-like strong transcriptional upregulation during the third larval instar. Their reduced abundance in starved hemolymph is therefore not a reflection of the inhibitory effect of starvation on development. Three of these gene models (*Npc2h*, *Tsf1*, *Pxn*) were previously found to be downregulated by starvation in adults according to transcriptomic analyses [78].

Apart from the 17 gene models characterized by reduced abundance of protein products in hemolymph of starved larvae, we detected 23 gene models with an opposite behavior (Table 2 and S2) using the stringent criteria described above. Judging from their developmental transcript profiles [80], increased product abundance in hemolymph from starved larvae in these cases is unlikely to be a secondary consequence of the inhibitory effect of starvation on development, with two possible exceptions, *Cpr51A* and *CG30457*. Moreover, in contrast to the proteins decreased in starved hemolymph, where a clear correlation was apparent with transcriptomics data from starvation experiments with adults [78], [79], this was not the case with proteins enriched in starved hemolymph. While starvation in adults was found to be accompanied by transcriptional downregulation of defense and immune response genes [78], our proteomics data from larval hemolymph did not reveal this same response. Several of the proteins enriched in starved hemolymph have actually been implicated in defense and immunity (Sp7, PO45, GNBP3, Spn55B, Spn77Ba). Apart from Spn55B and 77Ba, all other detected serpins (Spn4, Spn5, Spn27a, Spn88Eb, Spn43Ab, Spn28D, Spn1, Spn42E) except Spn1 appeared to be enriched in hemolymph from starved larvae as well, although with weaker statistical support. Serpins superfamily proteins are involved in the regulation of many different rapid physiological responses often by functioning as protease inhibitors [83]. However, given the small number of cases with robust statistical support, general conclusions concerning the effect of starvation onto defense and immune or any other process in larvae are impossible. Gene ontology analyses also failed to reveal statistically significant differences between the hemolymph proteomes of starved and fed larvae.

We would like to point out that our data should also be of considerable interest for further improvement of the *Drosophila* genome annotation, which so far is largely based on transcript analyses and a bias for long open reading frames.

### Conclusions

For high and middle abundance proteins, our study provides the first comprehensive picture of the composition of the hemolymph proteome in the *Drosophila* larva. Our data propel the known compositional complexity of *Drosophila* hemolymph more closely towards the state of the extensively characterized human plasma proteome. Our hemolymph proteome will support future data mining. The peptide catalogue (Table S1) can instruct future quantitative comparisons of the levels of hemolymph proteins in different developmental stages and physiological conditions using targeted proteomics approaches. Our initial comparison of hemolymph from fed and starved larvae by spectral counting indicated that the level of at least some proteins in *Drosophila* larval hemolymph is influenced dramatically by the nutritional status. Known storage proteins were far more abundant in hemolymph from fed compared to starved larvae. As the inhibitory effect of starvation on larval development appears to augment the primary response to nutritional status, our comparison points effectively to novel candidate storage proteins.

## Supporting Information

Table S1 **List of all identified peptides including characteristic properties and classification** [**38**] **.**
(XLS)

Table S2 **Complete data set (including identification of predicted small secreted globular proteins and comparison with previously published proteomic analyses of larval hemolymph).**
(XLS)

Table S3 **A listing of all identified Drosophila protein-groups (with their evidence class), ranked by differential expression (p-value calculated by DESeq).** Gene symbols and FlyBase IDs (“FBgn#”) are based on the mapping table from flybase.org. The spectral counts are shown for both conditions separately and for the combined total, followed by the DESeq normalized spectral counts that were used for the MA-plot (Figure 4). The log2-fold change is calculated based on the normalized spectral counts and the significance of differential expression is indicated by the DESeq p-value.
(XLS)
